# Altered social decision making in patients with chronic pain

**DOI:** 10.1017/S0033291721004359

**Published:** 2023-04

**Authors:** Alicja Timm, Tobias Schmidt-Wilcke, Sandra Blenk, Bettina Studer

**Affiliations:** 1 Medical Faculty, Institute of Clinical Neuroscience and Medical Psychology, Heinrich-Heine-University Düsseldorf, Düsseldorf, Germany; 2 Department of Neurology, Mauritius Hospital Meerbusch, Meerbusch, Germany; 3 Centre for Pain Medicine, St.Vinzenz Hospital Düsseldorf, Düsseldorf, Germany

**Keywords:** Social decision making, pain, inequity, unfairness, perceived injustice, cognition

## Abstract

**Background:**

Chronic pain affects up to 20% of the population, impairs quality of life and reduces social participation. Previous research reported that pain-related perceived injustice covaries with these negative consequences. The current study probed whether chronic pain patients responded more strongly to disadvantageous social inequity than healthy individuals.

**Methods:**

We administered the Ultimatum Game, a neuroeconomic social exchange game, where a sum of money is split between two players to a large sample of patients with chronic pain disorder with somatic and psychological factors (*n* = 102) and healthy controls (*n* = 101). Anonymised, and in truth experimentally controlled, co-players proposed a split, and our participants either accepted or rejected these offers.

**Results:**

Chronic pain patients were hypersensitive to disadvantageous inequity and punished their co-players for proposed unequal splits more often than healthy controls. Furthermore, this systematic shift in social decision making was independent of patients’ performance on tests of executive functions and risk-sensitive (non-social) decision making .

**Conclusions:**

Our findings indicate that chronic pain is associated with anomalies in social decision making (compared to healthy controls) and hypersensitivity to social inequity that is likely to negatively impact social partaking and thereby the quality of life.

## Introduction

Approximately 20% of adults suffer from chronic pain (Breivik, Collett, Ventafridda, Cohen, & Gallacher, 2006; Helmick et al., 2008; Johannes, Le, Zhou, Johnston, & Dworkin, 2010). Chronic pain severely impacts the quality of life (Breivik et al., 2006; Vartiainen, Heiskanen, Sintonen, Roine, & Kalso, 2016), reduces social participation (Baker, McBeth, Chew-Graham, & Wilkie, 2017; Simsek et al., 2010; Takeyachi et al., 2003), doubles suicide risk (Tang & Crane, 2006), and is frequently accompanied by depression (Knaster, Estlander, Karlsson, Kaprio, & Kalso, 2016; Miller & Cano, 2009; Serafini, Pryce, & Zachariou, 2020) and anhedonia (Garland, Trøstheim, Eikemo, Ernst, & Leknes, 2019). Chronification of pain is thought to result from a dynamic interaction between inadequate neural pain inhibition, maladaptive neuroplasticity, and psychosocial factors, including anxiety and lack of social support (Borsook, Youssef, Simons, Elman, & Eccleston, 2018; Ingvar, 2015; Morlion et al., 2018; Ossipov, Morimura, & Porreca, 2014). Extant explanations for the reduced social partaking often observed in patients suffering from chronic pain focus on pain-related disability, pain catastrophising, and depression (Ahlstrand, Björk, Thyberg, Börsbo, & Falkmer, 2012; Farin, 2015; Kim, Williams, Hassett, & Kratz, 2019). Other plausible mechanisms, e.g. that chronic pain affects social cognition, which in turn could impact social interactions, are discussed less frequently and have not been investigated systematically.

While deficits in executive functions have repeatedly been demonstrated in chronic pain patients (Apkarian et al., 2004; Attridge, Pickering, Inglis, Keogh, & Eccleston, 2019b; Glass, 2009; Hess, Haimovici, Muñoz, & Montoya, 2014; Luerding, Weigand, Bogdahn, & Schmidt-Wilcke, 2008; Suhr & Seng, 2012; Tamburin et al., 2014), social cognitions have received far less attention in previous research on the cognitive−affective effects of chronic pain. First studies did however find impairments in empathy (Sohn et al., 2016) and in the recognition of other people's emotions and mental states (Di Tella et al., 2015; Shin et al., 2013). Experimental acute pain has moreover been shown to alter moral judgments (Xiao, Zhu, & Luo, 2015) and interpersonal trust (Wang, Gao, Ma, Zhu, & Dong, 2018), and to reduce sharing with others (Mancini, Betti, Panasiti, Pavone, & Aglioti, 2011) as well as guilt over socially excluding or rejecting others (Bastian, Jetten, & Fasoli, 2011). A separate body of literature suggests that evaluations of social fairness impact the physical, mental, and psychosocial health of patients suffering from acute and chronic pain. Cross-sectional questionnaire studies found that the subjective experience of injustice related to the severity, blame, and irreparability of injury- and pain-induced loss is associated with more intense pain, higher physical disability, symptoms of depression, lower life satisfaction, and lower social functioning (see recent reviews by Carriere, Donayre Pimentel, Yakobov, & Edwards, 2020; Lynch, Fox, D'Alton, & Gaynor, 2021). Qualitative research confirms that chronic pain patients often feel treated unfairly and marginalised by family, friends, employers, colleagues, and the society (McParland, Eccleston, Osborn, & Hezseltine, 2010). A limitation of these findings is that self-reported perceived injustice may reflect both the objective injustices engraved in our society as well as potential pain-induced changes in fairness judgments. To the best of our knowledge, no study to date has assessed perceptions and responses of chronic pain patients to social inequity in an experimentally controlled setting.

The main goal of the current study was to probe whether chronic pain patients are more sensitive and respond more aversively to experimentally controlled disadvantageous social inequity than healthy individuals. Therefore, we administered a well-established neuroeconomic social exchange paradigm known as the Ultimatum Game (UG; Güth, Schmittberger, & Schwarze, 1982) to a large sample of chronic pain patients (*n* = 102) and matched healthy controls (*n* = 101). In this two-player game, one player (‘the proposer’) splits a sum of money between themselves and the other player (‘the responder’), who in turn decides to accept or reject the split offer. In case of a rejection, neither player gets any money. All participants were assigned to the responder role, and, unbeknownst to them, the proposer was computer-controlled and the proposed offers were predetermined. For the responder, the most lucrative choice is to accept all offers larger than zero. However, a multitude of previous studies have shown that offers with too large inequity in the split are perceived as unfair and rejected by most individuals (see review by Güth & Kocher, 2014). Such costly rejections, where own rewards are given up in order to punish the proposer of an unfair offer, demonstrate an affectively flavoured aversion against inequity (‘inequity aversion’, e.g. Fehr & Schmidt, 1999; Oberliessen & Kalenscher, 2019). We predicted that this inequity aversion would be heightened in chronic pain patients compared to healthy controls, reflecting a hypothesised increased sensitivity to unfair treatment by social interaction partners.

In addition to the UG, our participants completed a non-social decision-making paradigm called the Roulette Betting Task (RBT; Studer, Apergis-Schoute, Robbins, & Clark, 2012; Studer, Manes, Humphreys, Robbins, & Clark, 2015). In this task, participants decide how much money to place on risky gambles. Like the UG, it requires subjective valuation of the available options, but without the need to integrate social decision parameters. Use of the RBT thus allowed us to investigate whether value-based non-social choices were also altered in our sample of chronic pain patients, and therefore delignate whether observed differences in UG decisions may arise from a domain-independent shift in valuation processes, rather than the hypothesised hypersensitivity to disadvantageous social inequity.

## Methods

### Sample

One hundred and two chronic pain patients (27 male; Age: *M* = 49.75 years, s.d. = 14.64) and 101 healthy controls (34 male; Age: *M* = 40.35 years, s.d. = 15.08) participated in this study. Chronic pain patients were recruited from the outpatient Pain Clinic at the St. Vinzenz Hospital in Düsseldorf, where they received interdisciplinary treatment based on the biopsychosocial model. Healthy controls were recruited through advertisement at the University Düsseldorf and the Verbund Katholischer Kliniken Düsseldorf. All participants received a monetary pay-out based on one randomly selected trial of the UG. Healthy controls were additionally reimbursed a fixed rate of €80 for their participation in the larger protocol.

To further characterise our chronic pain patients, we collected self-reported average and current pain levels on a scale ranging from 0 to 10, scores on the Pain Disability Index (Tait, Chibnall, & Krause, 1990), a 7-item self-report instrument that assesses the degree to which chronic pain impairs daily activities, and scores on the Pain Catastrophising Scale (Sullivan, Bishop, & Pivik, 1995), a 13-item questionnaire that assesses how much patients ruminate about, magnify and feel helpless in managing their pain. One chronic pain patient failed to answer the questionnaires.

### Ultimatum game

**Fig. 1.**
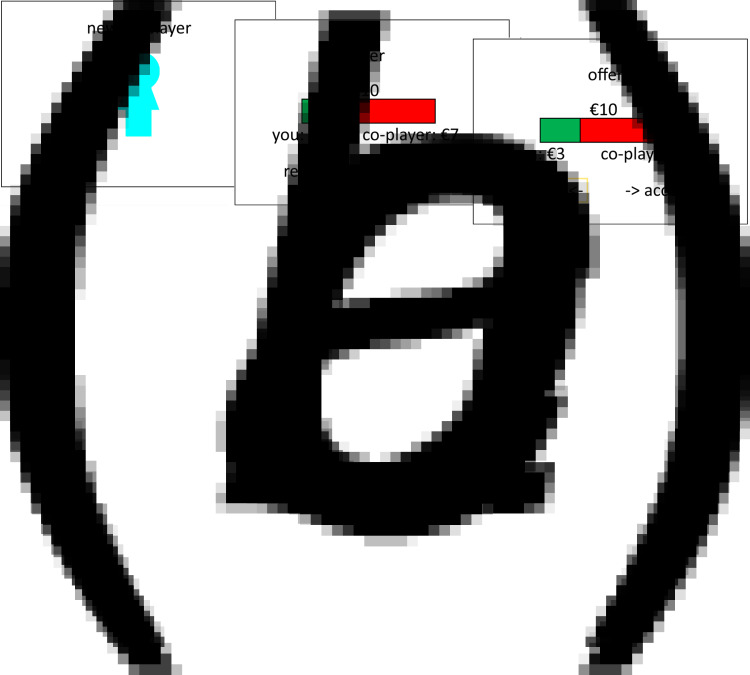
Ultimatum game. The figure shows an exemplary trial of the UG where the participant is first informed that they will play with a new co-player (*a*), then receives an offer with a €3:€7 split (*b*) and decides whether to accept or reject this offer. In the displayed example, the participant chooses to reject the offer (*c*; box added for visualisation).

We used an iterated version of the one-shot UG, where participants played against a new, gender-matched, anonym opponent on each trial (see Fig. 1). Participants were told that they would be connected to an online platform with a large pool of opponents and randomly assigned to the proposer or responder role at the beginning of the game. In truth, all participants played the UG in the role of the responder and presented offers were predetermined. The amount to be split was €10 in all cases, the offer size varied from €0:€10 to €5:€5, in one euro steps, across trials. The instructions further emphasised that they would play the game anonymously and would get assigned a new opponent in each round. This design allowed us to repeatedly sample participants’ responses to a specific offer while avoiding meta-cognitive influences that may occur in repeated interactions with the same co-player and implicit biases that may occur when playing against non-anonymised opponents (Mendoza, Lane, & Amodio, 2014; Solnick & Schweitzer, 1999). Participants were also informed that one round would be randomly selected for effective pay-out based on their recorded choices at the end of the game. Participants completed 55 rounds in the role of the responder, which entailed five repetitions of the €0:€10 offer (included primarily to verify task comprehension) and 10 repetitions of each other offer size.

### Roulette betting task

**Fig. 2.**
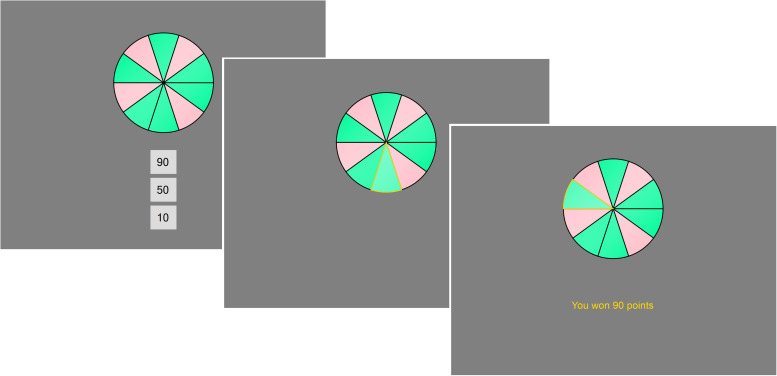
Roulette Betting Task. On this task, participants decide whether to bet 10, 50, or 90 points on a roulette-like gamble. Once a bet option is selected, the wheel spins and then lands on either a green segment (resulting in a win of the bet) or a red segment (resulting in a loss of the bet). Accumulated winnings were translated to a monetary pay-out at the end of the experiment.

The RBT (Studer & Clark, 2011; Studer et al., 2012, 2015) assesses risk-sensitive decision making. In each trial, a wheel with 10 blue (winning) and red (losing) segments and three bet options (10, 50, or 90 points) are presented (see Fig. 2). The chances of winning, determined by the ratio of green to red segments, were varied across trials (40, 50, 60, 70, and 80%). Participants first select a bet option through a corresponding keypress, then the wheel spins for a variable period (3000–6000 ms), and stops on one of the 10 segments. If the wheel stops on green, the bet is won; if it stopped on red, the bet is lost. Participants completed 5 practice bets, followed by 50 trials (10 repetitions for each odds level). Decision making was quantified through two calculated parameters reflecting (1) overall risk-taking (average bet amount) and (2) risk adjustment, formalised as the slope of the best line of fit through the average bet on the different chances levels. One patient and one healthy control failed to complete the RBT.

### Further measures

All participants took part in a larger protocol assessing cognitive and emotional deficits in chronic pain (see online Supplementary Material for details). The entire assessment lasted approximately 90 min including breaks, and was conducted in one or two sessions. We extracted four measures that allowed us to statistically control for cognitive and affective factors that could influence and confound choices on the UG from this larger assessment: two tests of executive functioning (the Go-No-Go Task and the Tower of London), one depression questionnaire (Becks Depression Inventory; Beck, Steer, & Brown, 1996), and one fatigue questionnaire (Fatigue Severity Scale; Krupp, LaRocca, Muir-Nash, & Steinberg, 1989). Executive functions, including behavioural inhibition and problem-solving, have sometimes been found to be impaired in chronic pain samples (Bell et al., 2018; Berryman et al., 2014; Cherry et al., 2014; Glass, 2009; Kerns, Rosenberg, & Otis, 2002; Solberg Nes, Roach, & Segerstrom, 2009) and linked to pain sensitivity and pain disability (Bjekić, Živanović, Purić, Oosterman, & Filipović, 2018; Elkana et al., 2020; Zhou, Kemp, Després, Pebayle, & Dufour, 2015), and may affect performance on decision-making tasks (e.g. Dittrich & Johansen, 2013; Manes et al., 2002). The Go-No-Go task (Drewe, 1975) measures the ability to control behavioural impulses. On each trial, one of two symbols (a cross and a plus), requiring opposite responses (a fast button press and no press, respectively) is displayed for 200 ms. The number of errors was extracted to quantify behavioural control and transformed to a percentage rank using established norms. The Tower of London task (ToL) assesses convergent problem solving. Three differently coloured balls arranged on three sticks are presented. The sticks are of different lengths and can hold a maximum of three, two, and one ball. The task is to rearrange the balls from a starting position to a predetermined end arrangement with as few moves as possible. The total number of moves needed was extracted as an outcome measure and transformed to percentage rank using established norms (Tucha & Lange, 2004).

### Ethical approval

This research was approved by the Ethics Committee of the Medical Faculty of the University of Düsseldorf, Germany (nr 201704466). All participants provided written informed consent. All procedures contributing to this work comply with the ethical standards of the relevant national and institutional committees on human experimentation and with the Helsinki Declaration of 1975, as revised in 2008.

### Statistical analyses

In order to compare social decision making of chronic pain patients and healthy controls, we first analysed accept/reject decisions on the UG with a repeated measures analyses of covariance (rANCOVA), with the factors offer (six levels), group (patients/controls), and offer × group interaction. Given that chronic pain patients were on average 9 years older than healthy controls (*t* = −4.48, *p* < 0.001), age was added as a covariate of no interest to this analysis. TOL performance, depression and fatigue scores were also added as covariates into this model, to statistically account for group differences in, and control for potentially confounding influences of, executive functioning and affective state. When the assumption of sphericity was violated, Greenhouse-Geisser corrections were applied. Differences in UG acceptance rates could result from a shift in equity aversion or simply from increased randomness in the choice behaviour. To investigate these two potential mechanisms, we ran a computational model over participants’ trial-by-trial decisions. This model provided two estimated parameters reflecting participants’ sensitivity to inequity (‘inequity aversion’, *i*) and their choice consistency (*β*, see section ‘Computational model of UG decisions’ for details), that were then compared between chronic pain patients and healthy controls with two separate ANCOVA (factor = group, covariates = age, depression scores, fatigue scores, TOL performance). Since sample distributions of *i* parameters were right-skewed (skewness_patients_ = 1.54, skewness_controls_ = 3.65), cube root transformation was applied prior to this analysis (see online Supplementary Fig. S1a). Additionally, Spearman correlations tested whether inequity aversion (transformed) and choice consistency were systematically related to current and average pain levels in the chronic pain group.

To test for potential differences in risk-sensitive decision making, overall betting and risk adjustment on the RBT were compared between chronic pain patients and healthy controls with ANCOVAs, again accounting for age, depression scores, fatigue scores and TOL performance. Since sample distributions of risk adjustment were left-skewed (skewness_patients_ = −1.60, skewness_controls_ = −1.28), the square transformation was applied prior to the ANCOVA (see online Supplementary Fig. S1b).

Our statistical analyses thus comprised two separate families of tests (one assessing social decision-making outcomes, the other assessing non-social decision-making outcomes). Within each of these families of tests, Bonferroni-corrections for multiple comparisons were applied and obtained *p*-values were compared to a corrected alpha level of 0.017 in the case of the group comparisons of the UG outcomes (three ANCOVAs) and of 0.025 in the case of the group comparisons of RBT outcomes (two ANCOVAs). Follow-up pairwise comparisons of acceptance rates at each offer level of the UG were likewise corrected for multiple comparisons. To allow readers to assess the statistical significance of effects with ease, we provide Bonferroni-corrected *p*-values using the denotation p_corr_ in the Results section.

All statistical analyses were computed in JASP and are reported two-sided.

#### Computational model of UG decisions

We modelled participants’ trial-by-trial reject/accept decisions on the UG as:
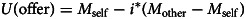
where *U*(offer) represents the utility of the proposed offer, *M*_self_ is the amount offered to the responder (i.e. 0, 1, 2, 3, 4 or 5 €), and *M*_other_ is the amount allocated to the proposer (i.e. 10, 8, 6, 4, 2 or 0 €), and the estimated parameter *i* (restricted to vary between 0 and 10) indicates how strongly the offer's utility is reduced by disadvantageous inequity (i.e. how much worse the proposer is off then the responder).^1^ When *i* = 0, the subjective value is equivalent to the amount offered to the responder and not affected by inequity. As *i* increases, responders become increasingly inequity averse, and the offer's utility is diminished and even becomes negative. Trial-by-trial utility estimates were transformed into a probability of offer acceptance using a softmax function:
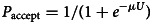
where *μ* is an inverse temperature parameter that characterises the choice consistency. The larger *μ*, the more consistent participants were in their reject/accept decisions for a given offer. Or vice versa, the smaller *μ*, the more varied was a participant's response to a given offer (see online Supplementary Fig. S2 for a visualisation).

## Results

### Characterisation of pain patients

All chronic pain patients underwent interdisciplinary outpatient treatment. Most (*n* = 84, 82%) suffered from chronic pain disorder with somatic and psychological factors (ICD-10-GM F45.41), characterised by persistent, severe, and distressing pain triggered by somatic disease but with causative influences from psychosocial factors. The remaining patients were diagnosed with dorsopathies (*n* = 6), gonarthrosis (*n* = 5), fibromyalgia (*n* = 3), polyneuropathy (*n* = 2), rheumatism (*n* = 1), and myalgia (*n* = 1). Chronic pain patients reported considerable levels of current and average pain (*M*_current_ = 5.96, SE_current_ = 0.24, *M*_average_ = 6.49, SE_average_ = 0.20) and pain-related disability (see Table 1), and the majority suffered from chronic pain for more than 2 years (> 5 years: 44%, 2–5 years: 20%, 1–2 years: 17%, 7–12 months: 11%, 1–6 months: 5%, <1 month: 2%). Chronic pain patients also had significantly higher depression and fatigue scores and performed worse on the ToL than healthy controls (see Table 1).

**Table 1.** Questionnaire scores and measures of executive functions

	Chronic pain patients (*n* = 101)			Healthy controls (*n* = 100)			Comparison	
	*M*	s.e.	Range	*M*	*M*	Range	*F*	*p*_corr_
Questionnaire scores								
Current pain intensity	5.96	0.24	[0, 10]	NA				
Average pain intensity	6.49	0.20	[0, 10]	NA				
Pain-related disability	35.88	2.59	[0, 100]	NA				
Pain catastrophising	25.60	1.27	[0, 52]	NA				
Depression	35.88	2.58	[0, 51]	4.64	0.67	[0, 43]	63.11	<0.001
Fatigue	4.63	0.16	[1, 7]	2.94	0.13	[1, 7]	62.50	<0.001
Executive functions								
Go-No-Go	21.63	2.27	[0, 66]	30.74	2.44	[0, 100]	4.00	0.09
Tower of London	29.44	3.14	[0, 99]	42.90	3.09	[0, 99]	8.47	0.008

*Note*: Questionnaire and executive function measures from one patient and one healthy control were missing.

### Chronic pain patients are more averse to social inequity

**Fig. 3.**
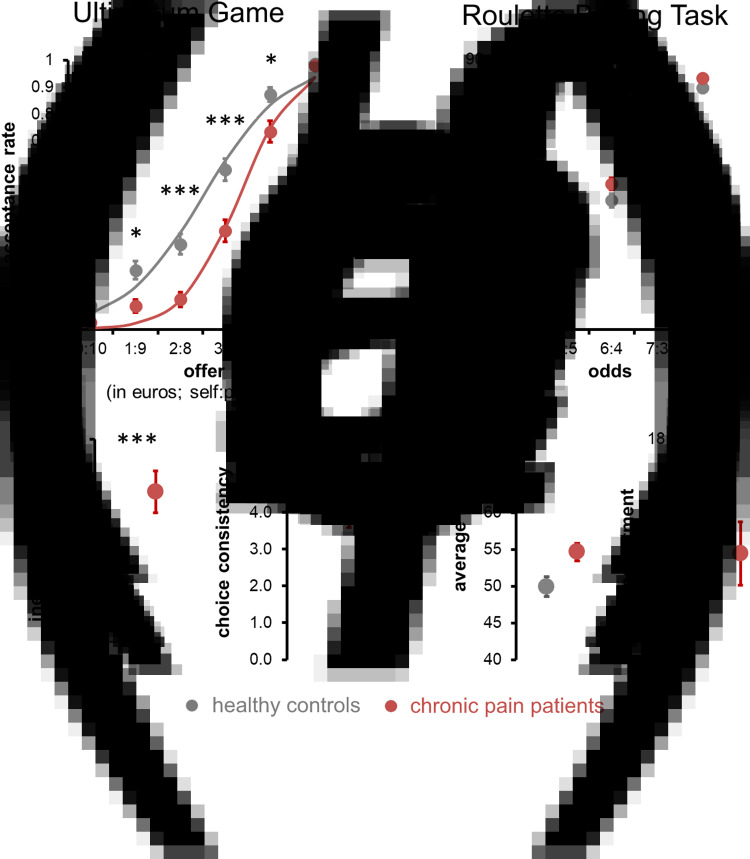
Social and risk-sensitive decision making. (*a*–*c*) Social decision making on the ultimatum game. (*a*) Average acceptance rates of UG offers in chronic pain patients (in red) and healthy controls (in grey). Dots represent observed sample means, the lines indicate model-predicted responses. (*b* and *c*) Estimated inequality aversion (*b*) was significantly higher in chronic pain patients (red) than healthy controls (grey), whereas estimated choice consistency (*c*) did not differ. (*d*–*f*) Risk-sensitivity decision making on RBT. (*d*) Bets were placed on the RBT as a function of the odds of winning (pain patients in red; healthy controls in grey). (*e*) Averaged across all odds levels, chronic pain patients placed higher bets than healthy controls, however, this group difference was not significant after controlling for depression scores. (*f*) Risk adjustment did not differ between chronic pain patients and healthy controls. Error bars represent s.e.m. ***p_corr_ < 0.001, **p_corr_ < 0.01, *p_corr_ < 0.05.

Observed and modelled acceptance rates of UG offers are displayed in Fig. 3*a*. The rANCOVA found a significant main effect of offer size, with the rejection rate increasing with higher inequity, *F*_(3.01/577.98)_ = 29.208, *p* < 0.0001, *ηp*^2^ = 0.130, a significant main effect of group, with chronic pain patients rejecting significantly more offers than healthy controls, *F*_(1/195)_ = 11.297, *p*_corr_ = 0.002, *ηp*^2^ = 0.055, *M*_pain_ = 62%, SE_pain_ = 1.8%, range_pain_ = [15%, 100%], *M*_controls_ = 49%, SE_controls_ = 2.1%, range_controls_ = [13%, 100%], and a significant offer × group interaction, *F*_(3.353/577.98)_ = 3.532, *p* = 0.015, *ηp*^2^ = 0.018. Follow-up level-wise *t* tests showed that healthy controls accepted significantly more offers with a 1:9, 2:8, 3:7 and 4:6 split than chronic pain patients, all *t*(199) ⩾ 2.957, *p*_corr_ ⩽ 0.021, whereas acceptance rates of offers with a 0:10 and 5:5 splits did not differ significantly between patients and controls, both *t*(199) ⩽ 1.30, *p*_corr_ = 1.00 (see Fig. 3*a* for mean rates and online Supplementary Table S1 for full statistics). No significant effects of age, depression scores, fatigue scores, or TOL performance were observed (all main effects: *F* ⩽ 1.124, *p* ⩾ 0.290, interactions with offer size: *F* ⩽ 0.103, *p* ⩾ 0.324, see online Supplementary Table S2 for full stats).

Model-based results showed that chronic pain patients were more inequity averse than healthy controls, *i* (untransformed): *M*_pain_ = 2.290, SE_pain_ = 0.289, range_pain_ = [0.001, 10.000], *M*_controls_ = 1.049, SE_controls_ = 0.174, range_pain_ = [0.001, 10.000], *i* (cube root transformed): *M*_pain_ = 1.113, SE_pain_ = 0.050, range_pain_ = [0.001, 2.154], *M*_controls_ = 0.844, SE_controls_ = 0.040, range_controls_ = [0.001, 2.154], *F*_(1/195)_ = 12.471, *p*_corr_ = 0.0001, *ηp*^2^ = 0.060, see Fig. 3*b*. Choice consistency did not significantly differ between patients and healthy controls, *β*: *M*_pain_ = 3.98, SE_pain_ = 0.34, range_pain_ = [0.005, 16.381], *M*_controls_ = 4.37, SE_controls_ = 0.40, range_controls_ = [0.181, 16.381], *F*_(1/195)_ = 0.888, *p*_corr_ = 1.00, *ηp*^2^ = 0.002. Neither inequity aversion nor choice consistency was significantly influenced by depression scores, fatigue scores, TOL performance, or age (all *F* ⩽ 0.948, *p* ⩾ 0.331, see online Supplementary Table S3 for full stats) or systematically related to patients’ current or average pain levels (rho ⩽ 0.119, *p*_corr_ ⩾ 0.95, see online Supplementary Table S4 for full stats). Together, these model-based results corroborate that the increased offer rejection rate of chronic pain patients was due to a systematic shift in the subjective valuation of unequal offers, rather than increased randomness in their choices.

Observed betting responses on the RBT are shown in Fig. 3*d*. Neither average bet amounts (*M*_pain_ = 54.66, SE_pain_ = 1.186, range_pain_ = [26.80, 86.80], *M*_controls_ = 49.95, SE_controls_ = 1.354, range_controls_ = [14.00, 90.00]) nor risk adjustment (untransformed: *M*_pain_ = 15.93, SE_pain_ = 0.58, range_pain_ = [−10, 24], *M*_controls_ = 16.63, SE_controls_ = 0.55, range_controls_ = [0, 24], square-transformed: *M*_pain_ = 286.68, SE_pain_ = 14.24, range_pain_ = [0, 576], *M*_controls_ = 306.63, SE_controls_ = 14.96, range_control_ = [0, 576]) differed significantly between chronic pain patients and healthy controls, *F*_(1/193)_ = 1.611, *p*_corr_ = 0.412, *ηp*^2^ = 0.008 and *F*_(1/193)_ = 0.211, *p*_corr_ = 1, *ηp*^2^ = 0.001, respectively. No systematic influences of age, fatigue scores or TOL performance upon average betting or risk adjustment were found, all *F*_(1/193)_ ⩾ 1.42, *p* ⩾ 0.235 (see online Supplementary Table S5 for full stats). The degree of risk adjustment was lower in those with higher depression scores, *F*_(1/193)_ = 5.143, *p* = 0.024, *ηp*^2^ = 0.026.

## Discussion

The results of our study indicate a systematic difference in the social decision making and social interaction behaviour of patients suffering from chronic pain and healthy individuals. Chronic pain patients rejected unequal offers on the UG significantly more often than healthy controls, displaying an inflated aversion to disadvantageous inequity and a higher willingness to forgo own monetary winnings to punish their opponents for unfair offers. Prior investigations found patients with fibromyalgia and chronic regional pain syndrome to be less accurate in recognising affective mental states on photos of human eyes (Di Tella et al., 2015; Shin et al., 2013) and to have a weaker self-reported empathic ability (Sohn et al., 2016). Our novel findings show that anomalies in the social cognitions of chronic pain patients are not limited to the (passive) recognition of other people's emotions, but rather also extend to actively respond to social interaction partners.

Social withdrawal is often observed in chronic pain patients (Baker et al., 2017; Simsek et al., 2010; Takeyachi et al., 2003) and is known to reduce the quality of life and life satisfaction (Ekström, Ivanoff, & Elmståhl, 2008). To date, reduced social participation has primarily been attributed to pain-related functional impairment and depression (Ahlstrand et al., 2012; Farin, 2015; Kim et al., 2019). But, alterations in interaction behaviour and social cognitions could also be a contributing factor. Inequity aversion acts as an unfairness detector that protects us from social exploitation (Brosnan & de Waal, 2014; Oberliessen & Kalenscher, 2019). However, hypersensitivity to inequity and perceived unfairness is likely to impede the quality of social interactions. Rejecting an unequal offer on the UG punishes the co-player and could even be interpreted as a provocation if the proposer considers the offer to be fair. Also, the responder has to forgo their monetary win. Overzealous rejection is thus both monetarily and socially costly. Likewise, in everyday life, a hypersensitivity to (perceived) unfairness of interaction partners is likely to reduce own pro-social behaviour and foster social isolation.

Our findings also critically extend the existing literature on perceived injustice and pain outcomes. This growing body of research suggests that questionnaire scores of perceived injustice related to the origin and consequences of acute and chronic pain are correlated with a higher disability, greater pain severity, lower social functions, and a higher prevalence of depression (e.g. Scott & Sullivan, 2012; Sturgeon et al., 2016; Sturgeon, Ziadni, Trost, Darnall, & Mackey, 2017; Sullivan, Scott, & Trost, 2012; Trost et al., 2019; Yakobov et al., 2014). In their recent reviews, Carriere et al. (2020) and Lynch et al. (2021) highlight that the self-report measure used in almost all of this research, the injustice experiences questionnaire (Sullivan et al., 2008), is likely to confound subjective perceptions of injustices and objective injustices (the prevalence of which may vary considerably across individuals with chronic pain). In the current study, we demonstrate that chronic pain patients respond stronger to experimentally controlled, i.e. objectively matched, social inequity than healthy controls, avoiding this pitfall. Secondly, our results show – for the first time - that fairness-related judgments *unrelated to the chronic pain condition itself* are also systematically altered in chronic pain patients compared to those observed in healthy controls. This is relevant to the question of the nature of the relationship between perceived injustice and health outcomes. While previous studies are almost exclusively correlational in nature and lack a guiding conceptual framework, the dominant view in the literature is that injustice perceptions arise from experienced violations of human rights, equity norms or just word beliefs (McParland & Eccleston, 2013; McParland, Knussen, & Murray, 2016; Monden, Trost, Scott, Bogart, & Driver, 2016) and are an antecedent of depressive symptoms and pain chronification (Lynch et al., 2021). We were not able to assess the pre-morbid social preferences of our patients. However, we reason that a shift in fairness judgments and responses to social inequity could plausibly also be a cognitive−affective consequence of chronic pain itself or of the systematic changes in brain function observed in chronic pain. Pain and social inequity are both thought to activate our alarm system to protect us from physical harm and social exploitation, respectively. And, there is some tentative evidence that activation of this system by one of these two factors may increase vigilance or sensitivity to the other as well. In healthy volunteers, sensitivity to physical pain increases after an experimentally induced experience of disadvantageous social inequity (Zhang, Wang, Sun, & Xie, 2021, experiment 3) and of social rejection (Eisenberger, Jarcho, Lieberman, & Naliboff, 2006) as well as following recall of an experience injustice (McParland et al., 2016). While an effect in the opposite direction has not yet been tested, it is plausible that the pain that our patients experienced during testing (see Table 1) increased their vigilance for social equity too, and thereby made them respond more strongly to it. Given that individual differences in pain sensitivity have been shown to correlate with rejection rates in the UG in healthy subjects (Wang, Li, & Xie, 2019, experiment 3) and since pain sensitivity is increased in chronic pain patients (e.g. Compton, Wasser, & Cheatle, 2020; Ruscheweyh et al., 2012), a complementary explanation could be that vigilance to social inequity is tonically increased in chronic pain patients as compared to healthy controls, independent of the concurrent pain experience.

Consistent with the idea that chronic pain may affect the processing of other information, systematic changes in the activation and functional connectivity patterns of multiple brain areas, including the insula, have been observed in chronic pain patients during cognitive tasks (e.g. Baliki, Geha, Apkarian, & Chialvo, 2008; Glass et al., 2011; Seminowicz et al., 2011; Weissman-Fogel et al., 2011). Functional neuroimaging studies of the UG in healthy volunteers also implicate the insula in responses to social inequity. Activation of the insula is stronger during the processing of unfair as compared to fair offers (Corradi-Dell'Acqua, Tusche, Vuilleumier, & Singer, 2016; Sanfey, Rilling, Aronson, Nystrom, & Cohen, 2003), and predicts offer rejection (Feng, Luo, & Krueger, 2015; Guo et al., 2013; Hollmann et al., 2011). And, effective connectivity between the insula and the anterior midcingulate cortex was linked to individuals’ reciprocity in a social interaction task (Shaw et al., 2019). Furthermore, lesions to the insula are associated with decreased inequity aversion (Nitsch, Strenger, Knecht, & Studer, 2021). As such, it is tempting to hypothesise that altered insular functioning in chronic pain could also play a role in the observed increased inequity aversion and rejection rates of unfair offers. Future studies might test this speculative link by concurrently assessing neural and behavioural responses to experimentally controlled social inequity in chronic pain patients.

Chronic pain patients’ and healthy controls’ decisions on the RBT did not differ systematically. This secondary decision-making task acted as a control task, assessing value-based choices outside of social context. While a lack of a statistically significant difference does not prove the absence of an effect, this finding nonetheless supports our conclusion that the increased rejection rates of chronic pain patients on the UG arose from hypersensitivity to inequity, rather than a domain-independent shift in valuation processes. In contrast, a handful of previous studies in chronic pain patients found poorer self-reported real-word decision outcomes (Attridge et al., 2019b) and impaired decision performance on the Iowa Gambling Task (IGT) (Apkarian et al., 2004; Hess et al., 2014; Tamburin et al., 2014; Verdejo-García, López-Torrecillas, Calandre, Delgado-Rodríguez, & Bechara, 2009). This divergence could be due to the differential cognitive processes required on these tasks, in particular, the need to learn reward/punishment contingencies through trial and error on the IGT, but not the RBT or the UG. This learning requirement makes IGT decisions more vulnerable to impairments in working memory (e.g. Bagneux, Thomassin, Gonthier, & Roulin, 2013; Ouerchefani, Ouerchefani, Allain, Ben Rejeb, & Le Gall, 2019), which have repeatedly been observed in chronic pain patients (Baker, Gibson, Georgiou-Karistianis, Roth, & Giummarra, 2016; Bell et al., 2018; Berryman et al., 2013; Mazza, Frot, & Rey, 2018). Consistently, a computational modelling study by Hess et al. (2014) found that IGT choices of chronic pain patients did not account for previous choices, unlike those of healthy controls, and Tamburin et al. (2014) found abnormal feedback processing and impaired learning in their chronic pain patients during the IGT.

A related alternative explanation worth discussing is whether the observed anomalies in our patients’ UG choices could have emerged from a general deficit in higher cognitive functions. Whereas previous research on the impact of experimental acute pain on higher cognitive functions and reasoning have yielded inconsistent results [see e.g. Attridge, Keogh, & Eccleston (2016, 2019a) *v.* Attridge et al. (2019b) and Pitães, Blais, Karoly, Okun, & Brewer (2018)], studies in patients with chronic pain have consistently found impairments in inhibitory control, set-shifting, planning, working memory, abstraction, and cognitive flexibility (Bell et al., 2018; Gunnarsson & Agerström, 2018; Munoz Ladron de Guevara, Fernandez-Serrano, Reyes Del Paso, & Duschek, 2018; Verdejo-García et al., 2009) [albeit, see Gunnarsson & Agerström (2021) for a null effect on logical reasoning]. Our sample of chronic pain patients indeed performed worse on the two executive function tasks than our healthy controls. However, scores on these executive function measures were not systematically related to rejections rates on the UG or model-estimated inequity aversion. Furthermore, our version of the UG provided all decision information in an explicit and intuitive way, required no integration or learning within or across trials, and was performed self-paced; requirements for working memory and other potentially confounding higher cognitive functions were therefore minimised. Finally, rather than systematically increasing rejection rates, a disruption in general processing abilities would arguably result in more erratic decision making. But, we found no evidence for a significant difference in choice consistency between our two groups. Together, our findings indicated that pain-related changes in social decision making are dissociable from other classically examined cognitive dysfunctions. Similarly, depression and fatigue scores were significantly higher in our chronic pain group than in healthy controls, but again these mood scores were not significantly related to rejection rates on the UG or inequity aversion. Depression scores were, however, significantly related to risk adjustment on the RBT, consistent with the rich literature on non-social decision-making deficits in patients with depression (for reviews, see Bishop & Gagne, 2018; Mukherjee & Kable, 2014).

There are two limitations to the current study: Firstly, our patients suffered from chronic pain of mixed aetiology. Future studies need to investigate pain subtypes, such as musculoskeletal pain, neuropathic pain, chronic headache, etc., to determine whether anomalies in social decision making and other social cognitions are specific to certain pain syndromes. Secondly, we focused on responder behaviour on the UG, which is a well-established approach to investigate behavioural, affective, and cognitive reactions to perceived unfairness in social exchanges. To complement our understanding of social interaction behaviour in chronic pain, future studies might investigate proposer behaviour on the UG in clinical samples.

In conclusion, this study indicates that social decision making differs systematically between chronic pain patients and healthy controls. Compared to our sample of healthy individuals, our chronic pain patients were hypersensitive to disadvantageous inequity in propositions made by their interaction partners. Given that socio-economical inequities are ubiquitous in our society and everyday life, such hypersensitivity is likely to impact social partaking and thereby might in the long term reduce quality of life . Social cognitions should become part of the neuropsychological diagnostics of chronic pain patients and potentially addressed by multidisciplinary pain therapy.

## Footnotes

1 This model is a simplified version of the seminal Fehr-Schmidt model of inequity aversion (Fehr & Schmidt, 1999), which additionally entails a utility loss from advantageous inequality (where the offer split favours the participant) – a case that did not occur in our design.

## Supplementary material

For supplementary material accompanying this paper visit https://doi.org/10.1017/S0033291721004359. click here to view supplementary material
