# Supplementary material for: Altered social decision making in patients with chronic pain
Source: Psychol Med. 2021 Nov 5;53(6):2466–75. doi: 10.1017/S0033291721004359 (PMC10123842; doi:10.1017/S0033291721004359)
Supplement: Supplementary file 1 [file S0033291721004359sup001.docx]

**SUPPLEMENTARY MATERIAL FOR**

**“Altered social decision-making in patients with chronic pain”**

by

Alicja Timm, Tobias Schmidt-Wilcke, Sandra Blenk, & Bettina Studer

**Group comparisons of UG inequity weighting and RBT risk adjustment following skew-reducing transformation**

The sample distributions of the inequity weighting parameter *ι* obtained for modelling UG responses and the risk adjustment (RA) parameter showed significant skew (skewness: *ι*_Patients_ = 1.54, *ι*_Controls_ = 3.65, RA_Patients_ = -1.60, RA_Controls_ = -1.28, kurtosis: *ι*_Patients_ = 0.843, *ι*_Controls_ = 13.54, RA_Patients_ = 3.60, RA_Controls_ = -1.16). Therefore, we applied cube root and square transformations to these measures, respectively (see Figure S1), and repeated all group comparisons reported in the main manuscript with the transformed data.

Results of these supplementary analyses were qualitatively identical to those found with the untransformed measures. Specifically, inequity aversion on the UG was significantly higher in chronic pain patients (F(1/198)=18.13, p_corr_ < .001, ηp^2^ = 0.084), whereas risk adjustment on the RBT did not differ significantly between chronic pain patients and healthy controls (F(1/196)=0.385, p_corr_ = 1, ηp^2^ = 0.003).

**Additional assessments of the larger protocol**

All participants of this study took part in a larger protocol assessing cognitive and emotional deficits in chronic pain, in which they were administered the following ten questionnaires:

- Pain Catastrophizing Scale (Sullivan *et al.*, 1995), which assesses whether patients overestimate their pain and are therefore prone to chronification with 13 questions each rated on a scale from 0 to 4 (total score range = 0 to 52).
- Fragebogen zur Erfassung der Schmerzverarbeitung (Geissner, 2001), which examines pain coping (cognitive and behavioral pain management) and pain-related psychological impairment (pain-related helplessness and depression, pain-related fear, pain-related anger) with 38 questions each rated on a scale from 1 to 6.
- Pain Disability Index (Chibnall and Tait, 1994), which assess subjective degree of chronic pain-related impairment of quality of life in seven areas (family and domestic obligations, recreation, social activities, work, sex life, self-sufficiency, essential activities), each rated on a scale ranging from 0 to 10.
- Becks Depression Inventory II (Kühner *et al.*, 2007), which tests for symptoms of depression with 21 questions each rated on a score from 0 to 3. Based on the number of points achieved, no depression (0-13), mild depression (14-19), moderate depression (20-28) and severe depression (29-63) can be distinguished.
- Fatigue Severity Scale Pain (Pfeffer, 2010).), which tests for chronic fatigue with 9 questions each rated on a scale from 1 to 7 (total score range = 0 to 63, mean score of 4 or larger indicates presence of fatigue).
- PainDetect (Nagel *et al.*, 2002), which screens for neuropathic pain components with 7 questions all rated on a scale from 0 to 5. Total scores range from 0 to 38 (total score < 13: neuropathic pain component unlikely; 13 – 18: unclear; > 18: neuropathic pain component likely).
- Conners Skalen für Verhalten und Aufmerksamkeit bei Erwachsenen (Christiansen *et al.*, 2012), which assesses symptoms of ADHD (four categories: inattention & memory problems, hyperactivity & motor agitation, impulsivity & emotional lability, self-concept problems) in adults with 66 questions each rated on a scale from 0 to 3.
- Dysexecutive Questionnaire (Wilson *et al.*, 1997), a self-report assessment of impairments in executive functions with 20 questions each rated on a scale from 0 to 4 (total score range = 0 to 80).
- Fragebogen zu erlebten Defiziten der Aufmerksamkeit (Suslow *et al.*, 1998), which assesses experienced deficits in attention (distractibility, slowdown, fatigue) with 27 questions each rated on a scale from 1 to 5.
- Depression, Anxiety and Stress subscales from the Deutscher Schmerzfragebogen (Nagel (Nagel *et al.*, 2002), with 7 questions for each subscale, each rated from 0 to 3.

They were also administered two additional cognitive tasks:

- Testbatterie zur Aufmerksamkeitsprüfung (TAP), which assesses attention functions and took approximately 30 minutes to complete
- Verbal Learning and Memory Test (VLMT), which tests verbal short-term and long-term memory functions and took 20 minutes to complete.

Data from these additional measures was not used in the current study.

Questionnaires were completed independently prior to the cognitive testing session.


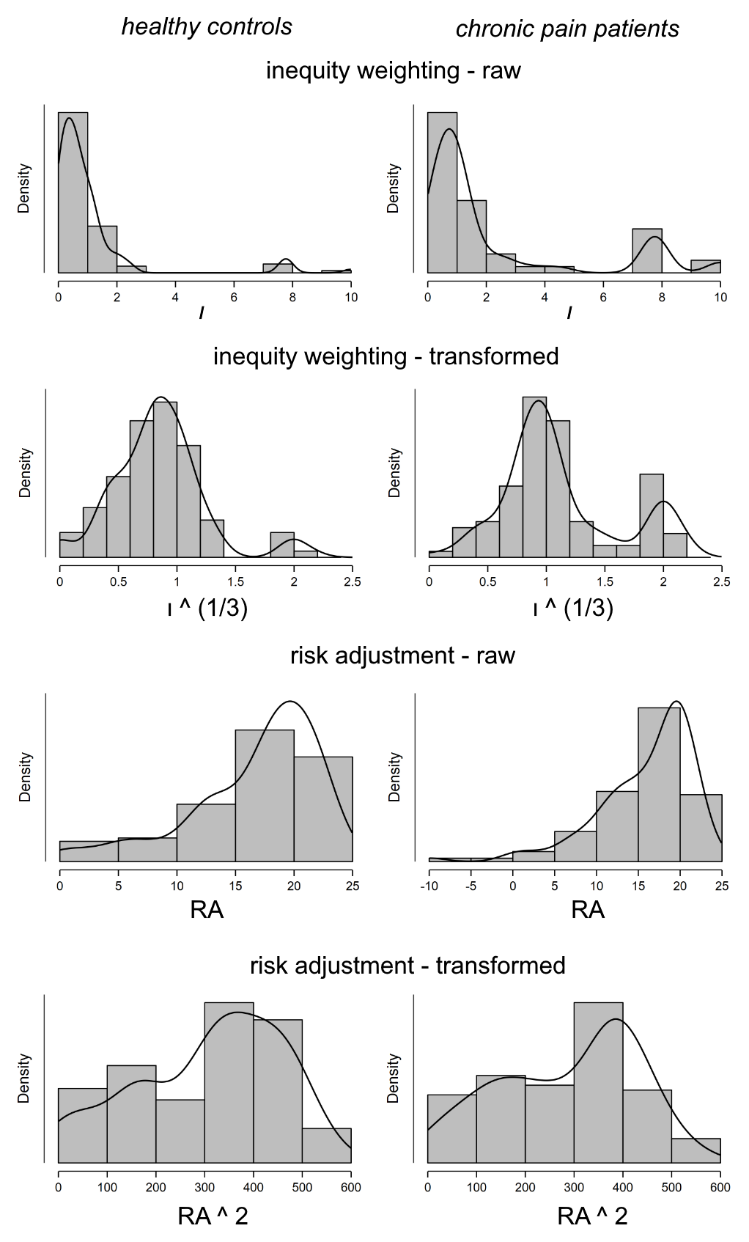


**Figure S1. Distribution plots of raw and transformed inequity weighting and risk adjustment measures**

The left column shows the distributions in healthy controls, the right shows the distributions in chronic pain patients.

**Figure S2 Visualisation of the impact of inequity aversion and choice consistency on UG responses**

*
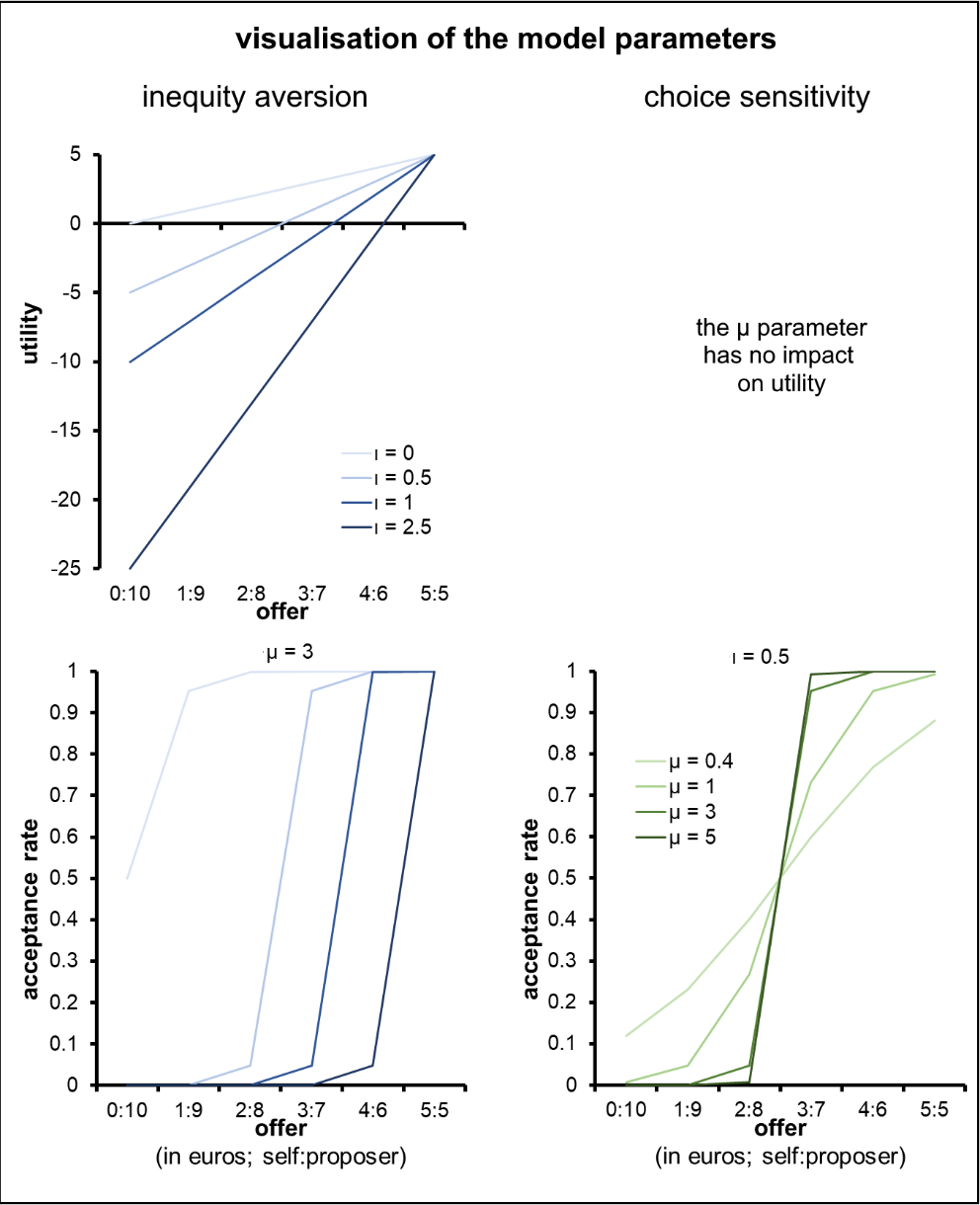
*

**Table S1.** Pair-wise comparisons of observed acceptance rates of chronic pain patients versus healthy controls at each offer size on the UG.

| offer | patients | | controls | | group comparison | | |
| --- | --- | --- | --- | --- | --- | --- | --- |
|  | mean | SE | mean | SE | t(199) | d | p_corr_ |
| 0€:1€ | 0.027 | 0.012 | 0.089 | 0.024 | -1.303 | 0.092 | 1.000 |
| **1€:9€** | **0.087** | **0.025** | **0.221** | **0.035** | **-2.957** | **0.209** | **.021** |
| **2€:8€** | **0.112** | **0.028** | **0.317** | **0.039** | **-4.367** | **0.308** | **< .001** |
| **3€:7€** | **0.367** | **0.041** | **0.594** | **0.042** | **-4.896** | **0.345** | **< .001** |
| **4€:6€** | **0.735** | **0.040** | **0.873** | **0.026** | **-2.981** | **0.210** | **.019** |
| 5€:5€ | 0.980 | 0.008 | 0.986 | 0.005 | -0.064 | 0.005 | 1.000 |

Note: Offers for which the group comparison was statistically significant are printed in bold.

**Table S2.** Results of rANCOVA on UG acceptance rate.

|  | F | p | η_p_² |
| --- | --- | --- | --- |
| offer | 29.208 | <.0001 | .130 |
| group | 11.297 | <.0001 | .055 |
| offer x group | 3.532 | .015 | .018 |
| age | 0.098 | .754 | .001 |
| depression scores | 1.021 | .314 | .005 |
| fatigue scores | 1.124 | .290 | .006 |
| TOL performance | 0.246 | .620 | .001 |
| offer x age | 0.292 | .829 | .001 |
| offer x depression scores | 0.508 | .674 | .003 |
| offer x fatigue scores | 1.161 | .324 | .006 |
| offer x TOL performance | 0.848 | .467 | .004 |

**Table S3.** Results of ANCOVAs on model-estimated inequity aversion and choice consistency.

|  | inequity aversion (transformed) | | | choice consistency | | |
| --- | --- | --- | --- | --- | --- | --- |
|  | F | p | η_p_² | F | p | η_p_² |
| group | 12.471 | <.0001 | .060 | 0.354 | .553 | .002 |
| age | 0.009 | .926 | .000 | 0.398 | .529 | .002 |
| depression scores | 1.213 | .272 | .006 | 0.948 | .331 | .005 |
| fatigue scores | 1.114 | .293 | .006 | 0.444 | .506 | .002 |
| TOL performance | 0.033 | .687 | .001 | <0.001 | .977 | .000 |

**Table S4.** Spearman correlations between model estimates of inequity aversion and choice consistency and current and average reported pain levels of chronic pain patients.

|  | current pain | average pain |
| --- | --- | --- |
| inequity aversion (*ι*) | rho = .110, p_corr_ = 1.00 | rho = .071, p_corr_ = 1.00 |
| choice consistency (*μ)* | rho = -.119, p_corr_ = 0.95 | rho = -.110, p_corr_ = 1.00 |

**Table S5.** Results of ANCOVAs on average bet and risk adjustment on the Roulette Betting Task (RBT)

|  | average bet | | | risk adjustment  (transformed) | | |
| --- | --- | --- | --- | --- | --- | --- |
|  | F | p | η_p_² | F | p | η_p_² |
| group | 1.611 | .206 | .008 | 0.048 | .827 | .000 |
| age | 0.253 | .615 | .001 | 0.373 | .542 | .002 |
| depression scores | 0.864 | .354 | .004 | 5.143 | .024 | .026 |
| fatigue scores | 0.003 | .955 | .000 | 1.069 | .302 | .006 |
| TOL performance | 0.377 | .540 | .002 | 1.420 | .235 | .007 |

**Table S6.** Reliability index for the questionnaire measures

|  | Cronbachs α |
| --- | --- |
| Becks Depression Inventory | .943 |
| Fatigue Severity Scale | .947 |
| Pain Disability Index | .888 |
| Pain Catastrophizing Scale | .931 |

**References**

Chibnall, J. T. & Tait, R. C. (1994). The Pain Disability Index: factor structure and normative data. *Archives of Physical Medicine Rehabilitation* 75, 1082-1086.

Christiansen, H., Kis, B., Hirsch, O., Matthies, S., Hebebrand, J., Uekermann, J., Abdel-Hamid, M., Kraemer, M., Wiltfang, J. & Graf, E. (2012). German validation of the Conners Adult ADHD Rating Scales (CAARS) II: Reliability, validity, diagnostic sensitivity and specificity. *European Psychiatry* 27, 321-328.

Geissner, E. (2001). *Fragebogen zur Erfassung der Schmerzverarbeitung: FESV*. Hogrefe, Verlag für Psychologie.

Kühner, C., Bürger, C., Keller, F. & Hautzinger, M. (2007). Reliabilität und Validität des revidierten Beck-Depressionsinventars (BDI-II). *Nervenarzt* 78, 651-656.

Nagel, B., Gerbershagen, H., Lindena, G. & Pfingsten, M. (2002). Entwicklung und empirische Überprüfung des Deutschen Schmerzfragebogens der DGSS. *Der Schmerz* 16, 263-270.

Pfeffer, A. (2010). Assessment: Fatigue Severity Scale – Einsatz bei Erschöpfung. *Ergopraxis* 3, 26-27.

Sullivan, M. J., Bishop, S. R. & Pivik, J. (1995). The Pain Catastrophizing Scale: Development and Validation. *Psychological Assessment* 7, 524.

Suslow, T., Arolt, V. & Junghanns, K. (1998). Differentielle Validität des Fragebogen erlebter Defizite der Aufmerksamkeit (FEDA): konkurrente Validierungsergebnisse bei Schizophrenen und Depressiven Patienten. *Zeitschrift für Klinische Psychologie, Psychiatrie und Psychotherapie*.

Wilson, B. A., Evans, J. J., Alderman, N., Burgess, P. W. & Emslie, H. (1997). Behavioural Assessment of the Dysexecutive Syndrome. *Methodology of frontal executive function* 239, 250.
